# Systematic review of neuroimaging findings in children and young adults with chronic kidney disease

**DOI:** 10.1007/s00467-025-07094-5

**Published:** 2025-12-11

**Authors:** Promesse Kayumba, Ryan Ward, Rodney D. Gilbert, Lyndsay Harshman, Matthew Harmer

**Affiliations:** 1 University of Southampton, Southampton, UK; 2 Stead Family Department of Paediatrics, University of Iowa Carver College of Medicine, Iowa City, IA USA; 3 Southampton Children’s Hospital, Southampton, UK; 4 Department of Paediatric Nephrology, G-level, Southampton Children’s Hospital, University Hospital Southampton NHS Foundation Trust, Tremona Road, Southampton, SO16 6YD United Kingdom

**Keywords:** CKD, EGFR, Neurodevelopment, Neuroimaging

## Abstract

**Background:**

Chronic kidney disease (CKD) is associated with poorer neurocognitive outcomes. Neuroimaging studies have been mostly undertaken in older adults. However, individuals under 25 are uniquely impacted due to concurrent brain development.

**Aims:**

This systematic review explored neuroimaging in children and young adults with CKD aiming to characterize brain outcomes in paediatric CKD.

**Methods:**

A systematic search of MEDLINE Ovid, EMBASE and Cochrane databases was undertaken on October 10, 2024. Observational studies involving patients with CKD aged 0–25 years and having neuroimaging findings as a key outcome were included. Following quality assessment using the Newcastle–Ottawa scale, a narrative review was performed.

**Results:**

Sixteen studies were included: 737 individuals across studies. Smaller brain volumes were reported in three of five studies (with one contradictory). White matter integrity was impaired, even in patients with mild disease. The latter was associated with a lower intelligence quotient. Silent brain infarcts were identified in up to 79% of dialysis patients with associations with markers of bone disease, longer dialysis duration and haemodynamic instability. Brain regions involved in attention, executive functions and the resting-state network demonstrated abnormal connectivity that was associated with longer reactivity and a response time in visual memory task. Measures of anaemia were inversely associated with increased cerebral blood flow (CBF) which corresponded with poorer verbal memory.

**Conclusion:**

Neurodevelopment is altered in paediatric CKD. Although a global mechanism for this remains unclear, it is evident that even early CKD is associated with increased risk of brain damage and cognitive deficits. Treatment methods and duration, disease severity and blood calcium and parathyroid concentrations were strongly associated with brain injury. Future work should prioritize longitudinal models of assessment with a focus on recruitment of uniform groups based on age, disease severity, aetiology and treatment to more clearly delineate the effects of disease on neurodevelopment.

**Graphical Abstract:**

**Figure:**
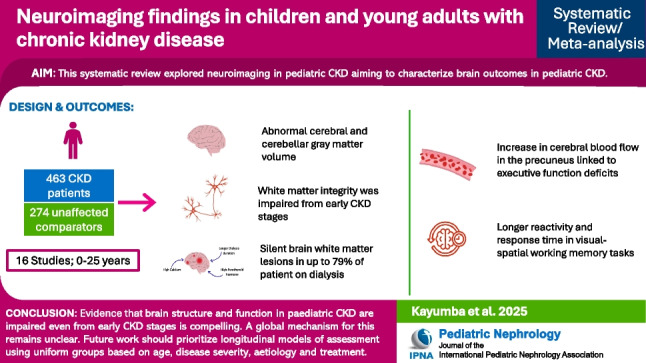
A higher resolution version of the Graphical abstract is available as Supplementary information.

**Supplementary Information:**

The online version contains supplementary material available at 10.1007/s00467-025-07094-5.

## Introduction

Paediatric chronic kidney disease (CKD) confers a significant risk factor for neurocognitive impairment throughout childhood and into adulthood [1–5]. Even in early stages of disease [2, 3], children with CKD demonstrate deficits in executive functioning (EF), with attention and working memory being particularly affected [2, 3, 5–7]. These impairments extend beyond cognitive domains to impact social behaviour [2, 5, 8], adaptive functioning [3, 5, 8, 9] and academic performance [10, 11], with lasting consequences for relationships [4, 11], future employment [11] and quality of life [3, 6–8, 12].

Theoretical models of neurocognitive development in illnesses [13] suggest a critical role between age of disease onset, disease severity, treatment and the psychological and emotional impact on the child and his family [13], although the causes and magnitude of those associations remain uncertain. Neurocognitive impairment in CKD has been associated with uraemia [14], anaemia [15], vitamin D deficiency [16], acidosis [17] and hypertension (HTN) [8], all of which are commonly observed in patients with CKD. Furthermore, CKD-associated treatments including dialysis and medications (e.g., immunosuppressants such as tacrolimus) can exert adverse neurological effects [18]. Despite modern approaches to care, including removal of aluminium-containing compounds for phosphorus binding, dialysis status remains associated with poorer neurocognitive outcomes [5, 19].

Neuroimaging offers tools to noninvasively and quantitatively evaluate both brain structure and brain function. For disorders that are primarily brain-based, neuroimaging has provided tremendous gains in understanding of abnormalities and mechanisms of disease. Adult CKD neuroimaging studies have documented cerebral atrophy, white matter hyperintensities and altered connectivity patterns associated with decreased kidney function [19, 20]. Lower kidney function and albuminuria also predict the presence of white matter hyperintensities, potentially reflecting silent brain infarcts (SBI) [19, 21]. Despite relatively robust adult literature on this topic, adult findings cannot be generalized to children and young adults given the dynamic neurodevelopmental changes occurring until the mid-20 s [22]. Lack of focus on early-stage CKD removes the ability to identify potentially modifiable, disease-based risk factors that may impact brain development in this life-long disease process. In fact, there is historically a skew to inclusion of more severe disease status—those requiring dialysis or transplantation—within CKD neuroimaging studies. There has been inclusion of widely variable disease aetiology in CKD samples evaluated to date, including both congenital disease and non-congenital disease (e.g., glomerular/immune in nature, such as lupus nephritis).

Given these gaps in the literature, this systematic review aimed to evaluate neuroimaging research on neurodevelopment in children and young adults with CKD, synthesizing evidence across all disease stages and imaging modalities.

This is the first review to apply clearly defined age cutoffs (0–25 years) to focus specifically on the developing brain, enhancing the relevance of our findings to the paediatric and young adult populations. It aims to draw clear and meaningful insights from studies that in themselves are limited by small sample sizes and wide heterogeneity in disease aetiology and treatment, reducing their capacity to find true effects. By synthesizing these studies, we seek to uncover key patterns or trends that may be hidden in smaller analyses. We also aim to explore the heterogeneity to generate hypotheses about the potential influence of specific variables.

## Methods

A Protocol was developed and registered with the PROSPERO registry following PRISMA-P guidelines (CRD42024554240) (see Appendix 1) [23, 24].

### Search strategy

A systematic search of MEDLINE Ovid, EMBASE and Cochrane databases was performed on October 10, 2024, using prespecified search terms. A comprehensive list with synonyms of ‘children’, ‘young adult’ and ‘brain’, with relevant neuroimaging methods, was used for a free word search and run alongside platform-specific thesauruses (Table S1. *Supplementary materials*). Limitations to English language, year 1980–2024 and exclusion of non-human studies using the Boolean operator “NOT” were applied. Studies examining rare genetic disorders associated with CKD were excluded as the aim of the review was to assess the general impact of CKD. The search results were imported into EndNote for deduplication then transferred to Rayyan [25] a systematic review management software for screening in two stages by two independent, blinded reviewers (PK and RW). A third reviewer (LH) resolved conflicts. The inclusion and exclusion criteria (Table S2. Supplementary materials) were systematically applied. A cutoff age of 25 years was applied to the mean age of studies to focus on CKD aetiologies particular to younger populations and to assess the effects of CKD during periods of ongoing brain maturation, which is commonly complete around 25 years of age. Stage 1 = title and abstract review, and stage 2 = full text review. Manuscripts categorized as “Maybe” and “Conflict” were reviewed with the third reviewer for final decision. No citations were lost.

### Data collection

Data collection by PK and verified by LH and RW, used a piloted, Microsoft Excel extraction table. If data were missing, authors were emailed. Data of study setting, participants, disease characteristics and key findings were extracted. Key findings were defined as outcomes examining the association between any brain imaging method and CKD or associated disease variables, e.g., kidney function/duration of disease. Associations to neurocognitive outcomes were extracted for clinical significance.

### Quality assessment

Risk of bias was evaluated using the Newcastle–Ottawa scales (NOS) [26, 27].

### Data synthesis

Extracted data were synthesized into Table 1. Given the heterogeneity of studies, a synthesis Without-Meta-Analysis was used, using the reporting items checklist [28]. Key findings were tabulated based on the overall intervention used, structural or functional brain imaging (Table 2). Data were further grouped by outcome.

**Table 1:** Included studies characteristics

Author	Country	Method	Participants	CKD status	Intervention	Outcomes studied	Funding
Carlström, 2016	Sweden	Longitudinal	*n* = 10 CKD patients; 3 male, 10 female; 6 infants, 4 children; median age 0.22 yr	Stage 5, all on peritoneal dialysis. Treatment method: peritoneal dialysis	Restrospective CT scans; T2WI and FLAIR MRI; DWI-MRI, ADC-MRI	General brain findings, plasma	Swedish Heart–Lung Foundation grants, Swedish Research Council, CERIC Linnaeus Grant, NovoNordisk Foundation Grant, Stockholm City Council(ALF), and by Karolinska Institutet Funding
Elzouki, 1994	Kuwait (Middle East)	Prospective	*n* = 15 CKD patients; 13 male, 2 female; mean age 4.5 yr. All had chronic renal disease at birth or during 1 st year of life	ESRD = 6 Px, CRF = 5 Px Stage 3–4 = 4 Px. Treatment method: dialysis, transplant	CT scans	Disease-related variables/severity, general brain findings	/
Harrell, 2021	USA (School of Medicine, University of North Carolina-Chapel Hill)	Cross-sectional with Control group	*n* = 21 CKD patients; 12 male, 9 female; mean age 14.4 yr. *n* = 11 HC; 6 male, 5 female; mean age 14.5 yr	Stage 2–3 = 10 Px and Stage 4–5 = 11 Px. Treatment method: conservative, dialysis, transplant	Structural T1WI-MRI. fMRI-BOLD on 3 T siemens magnetom. ROI analysis. MRI is done while participants performing a visuo-spatial working memory task. 2 study groups: CKD and HC	Activation pattern (BOLD), disease -related variables/severity	The Chronic Kidney disease in Children prospective cohort study (CKiD) which is funded by the National Institute of Diabetes and Digestive and Kidney Diseases, with additional funding from the National Institute of Child Health and Human Development, and the National Heart, Lung, and Blood Institute "
Hartung, 2018	USA (Children’s Hospital of Philadelphia)	NiCK cross-sectional cohort study with Control group	*n* = 82 CKD patient; 54 male and 28 female; mean age 16.3 yr. *n* = 63 HC; 33 male, 30 female; mean age 15.9 yr	Stage 2 to 5 with 52% at eGFR above or equal to 45 ml/min/1.73 m^2^ (Stage 3a and above). Treatment method: conservative, transplant. 33% had glomerular diagnosis	T1WI, T2WI, FLAIR, T1 FLASH and T2 proton-density MRI on Siemens Verio 3 T. ROI analysis. 2 study groups: CKD and HC	Brain volume, neurocognition, GM volume	Funding as part of the NiCK study project was partially done through Commonwealth Universal Research Enhancement grant with the Pennsylvania Department of Health. Additional support from National Center for Research Resources and the National Center for Advancing Translational Sciences, National Institutes of Health, through Grants
Herrington, 2021	USA	Cross-sectional with Control group	*n* = 67 CKD patients; 42 male, 25 female; mean age 16.8 yr. *n* = 58 HC; 28 male, 30 female; mean age 16 yr	No CKD staging; eGFR of CKD grp is 47.5 ml/min/1.73 m^2^ (SD = 24.1). Treatment method: conservative, dialysis, transplant	MRI volume. Resting state-fMRI-BOLD on Siemens Verio 3 T scanner. 2 study groups: CKD and HC	Activation pattern (BOLD), Disease-related variables/severity, functional connectivity	Commonwealth Universal Research Enhancement grant, the National Center for Advancing Translational Sciences of the National Institutes of Health grant, the National Center for Research Resources and the National Center for Advancing Translational Sciences, National Institutes of Health, and the National Center for Diabetes and Digestive and Kidney Disease, National Institutes of Health grant
Lijdsman, 2021	Netherlands	Cross-sectional with Control group	*n* = 24 CKD patients; 16 male, 8 female; median age 18.5 yr. *n* = 21 HC; 13 male, 8 female; median age 19.8 yr	Stage 4–5. Treatment method: conservative, haemodialysis, peritoneal dialysis, transplant	T1WI MRI, dMRI with DTI-TBSS. 2 study groups: CKD and HC	Brain volume, treatment modality, WM abnormalities	Partly funded by the Dutch Kidney Foundation
Lijdsman, 2022	Netherlands	Cross-sectional	*n* = 28 CKD patients; 18 male, 10 female; median age 18.5 yr	Stage 4–5. Treatment method: conservative, dialysis, transplant	T1WI MRI, DTI-TBSS, dMRI	Disease-related variables/severity, neurocognition, WM abnormalities. 3 groups: pre-dialysis, dialysis and transplant	Partly funded by the Dutch Kidney Foundation
Liu, 2018a	USA (Children’s Hospital of Philadelphia)	Cross-sectional with Control group	*n* = 61 CKD patients; 42 male, 19 female, mean age 14.67 yr. *n* = 47 HC; 23 male, 24 female; mean age 14.36 yr	Stage 2–5. Unknown treatment method	Structural MRI on 3 T whole-body Siemens Verio. fMRI-ASL using pseudocontinuous ASL. 2 study groups: CKD and HC	Cerebral blood flow	/
Liu, 2018b	USA (Children’s Hospital of Philadelphia)	NiCK cross-sectional cohort study with Control group	*n* = 73 CKD patients; 48 male, 25 female; mean age 15.8 yr. *n* = 57 HC; 29 male, 28 female; mean age 15.65 yr	Stage 2–5	Structural MRI, T2WI, FLAIR using 3 T whole body; fMRI-ASL using pseudocontinuous ASL. 2 study groups: CKD and HC	Cerebral blood flow, disease-related variables/severity, neurocognition	Commonwealth Universal Research Enhancement grant. National Center for Research Resources and the National Center for Advancing Translational Sciences, National Institutes of Health grants. Taipei Medical University Hospital grants and the Ministry of Science and Technology in Taiwan
Matsuda-Abedini, 2018	USA (North American Hospitals)	Cross-sectional with Control group	*n* = 29 CKD patient; 16 male and 13 female; of the CKD, 10 had RT; mean age 14.4 yr. *n* = 20 HC; 12 male and 8 female; mean age 13.7 yr	Stage 2, 3, 4 and 5 (with 8Px, 10Px, 4Px and 7Px respectively). Treatment method: conservative, peritoneal dialysis, transplant. CAKUT = 55%; glomerulonephritis = 45%	Conventional MRI at 2 different sites; dMRI with DTI-TBSS as well as ROI analysis. 3 study groups: CKD, RT and HC	WM abnormalities	British Columbia Children’s Hospital Telethon Foundation Grant, the Renal Research Institute at the University of North Carolina School of Medicine, and by NCTraCTS Institute at the University of North Carolina School of Medicine
Solomon, 2021	USA (University of Iowa Stead Family Children’s Hospital)	Cross-sectional with Control group	*n* = 18 CKD patients; all males; mean age 12 yr. *n* = 24 HC; all males; mean age 11.9 yr	Early CKD: Stage 1 to 3 (6 in each). Conservative treatment. All had CAKUT	T1WI and T2WI MRI on 2 different scanner: GEDiscovery 750 W 3 T and Siemens TrioTim 3 T; ROI analysis. 2 study groups: CKD and HC	Disease-related variables/severity, GM volume, neurocognition	National Institute of Diabetes and Digestive and Kidney Diseases
Steinberg, 1985	Jerusalem (Middle East)	Cross-sectional, Retrospective	*n* = 22 CKD patients; 12 male, 10 female, mean age 10.22 yr	CRF (stage in which the residual function is < 25% of normal ~ Stage 4). Treatment method: conservative, haemodialysis, peritoneal dialysis, transplant	Non-contrast CT scans	General brain findings	/
Valanne, 2004	Finland (Helsinki University)	Cross-sectional, with some retrospective data	*n* = 33 CKD patients; 22 male, 11 female; mean age 8 yr. All underwent renal transplant before 5 years of age; study is done at a mean of 6 yr post-transplant	CRF (stage in which the residual function is < 25% of normal ~ Stage 4). Transplant. 88% had CNS of Finnish type	CT scans, conventional MRI	General brain findings, ischemic lesions	/
van der Plas, 2022a	USA	Cross-sectional with Control group	*n* = 17 CKD patients; all male, mean age 12y. *n* = 20 HC; all male, mean age 11.9 yr	Stage 1–3. Conservative treatment. All have CAKUT	T1WI and T2WI non-sedated, non-contrast MRI on 3 T scanner. dMRI. 2 study groups: CKD and HC	Disease-related variables/severity, neurocognition, WM abnormalities	National Institute of Diabetes and Digestive and Kidney Diseases
van der Plas, 2022b	USA (University of Iowa)	Cross-sectional with Control group	*n* = 16 CKD; patients; all male; mean age 11.9 yr. *n* = 23 HC; all male; mean age 12 yr	Stage 2–3. Treatment method: conservative. All have CAKUT	T1WI and T2WI MRI on 2 different scanner: GEDiscovery 750 W 3 T and Siemens TrioTim 3 T	Disease-related variables/severity; brain volume. 2 study groups: CKD and HC	National Institute of Diabetes and Digestive and Kidney Diseases
Zaki, 2021	Egypt (Suez Canal University, Middle East)	Cross-sectional	*n* = 48 CKD patients; 26 male, 22 female; mean age 19.6 yr	ESRD. Treatment method: haemodialysis	T1WI, T2WI, FLAIR and SWI MRI on 1.3 T	Ischemic lesions, microbleeds, WM abnormalities; Disease-related variables/severity	/

*Px* patients, *CKD* chronic kidney disease, *HC* health controls, *CAKUT* congenital anomalies of the kidney and urinary tract, *ESRD* end-stage renal disease, *CRF* chronic renal failure, *MRI* magnetic resonance imaging, *WI* weighted images, *FLAIR* fluid-attenuated inversion recovery, *FLASH* fast low angle shot, *CT* computerise tomography, *dMRI* diffusion MRI, *SWI* susceptibility weighted imaging, *DTI* diffusion tensor imaging, *TBSS* tract-based statistical analysis, *ADC* apparent diffusion coefficient, *fMRI* functional MRI, *BOLD* blood-oxygen level dependent, *ASL* arterial spin labelling, *GM* gray matter, *WM* white matter, *ROI* regions of interest, *NiCK* Neurocognitive Assessment and Magnetic Resonance Imaging Analysis of Children and Young Adults with Chronic Kidney Disease

**Table 2:** Summary of key study findings

Study ID	Risk of bias	Sample	Imaging
**Structural**			
Carlström, 2016	High	Infants and children on PD with median follow-up of 9 months (range 3 − 22 months)	Serial neuroimaging study in infants and children on PD alongside nitrate, nitrite and BP measurement. Px 1 to 3 had systemic low SBP (< 50th percentile for age). In patients that started PD as neonates, 67% (*n* = 4) had abnormal neuroimaging (Px 1,2,3,5). Px 3: pseudocyst, gliosis and multi-cystic encephalomalacia affecting every lobes. Px 1 and 2 had encephalomalacia in the occipital lobe. Px 5 had bilateral infarctions in the parietal lobe and thalamus following a septic event. In the post-infancy PD patients 67% (*n* = 2) had abnormal findings. Px 10 had cerebral atrophy. Px 9: gliosis in periventricular WM regions and cortical encephalomalacia in left parietal lobe. Only 1 Px showed neurologic deficits identified as slightly retarded motor development, and mild hypotonia. Post-PD sessions showed across all Px: loss of plasma nitrite (mean = − 34 ± 4%, *p* < 0.05), inorganic nitrate (mean = − 17 ± 3%, *p* < 0.05) and cGMP levels (mean = − 59.4 ± 15%, *p* = 0.006)
Elzouki, 1994	Very high	CRF/ESRD and Mild-moderate (dialysis, RT, unknown)	CT scan was performed after a no oral aluminium salts and early nutritional and psychosocial support intervention. Cerebral atrophy was present only 3 patients (23%); with 2 classified as mild and 1 as moderate. Microcephaly was found in 5 patients (38%) including 3 who also had developmental delay. Malnutrition in the first 2 years of life was significantly correlated to microcephaly (*p* < 0.005). 6 Px had Abnormal EEG results. 1 Px had diminished nerve conduction velocity. 4 Px had increased polyphasia of motor unit potentials measured by EMG. 67% of Px had hypotonia
Hartung, 2018^‡^	Low	NiCK study cohort CKD Stage 2 − 5 vs. HC and subgroup analysis: RT vs. non-RT	Structural brain MRI in 19 ROIs in 7 domains. No statistically significant results were found only meaningful trends: CKD compared to HC had lower volumes in WB, cortex and left parietal GM (*p* = 0.08 for all). WML were present in 7% (*n* = 6) of the CKD participants vs. 3% (*n* = 2) of the HC (*p* = 0.5). No differences were found in other ROIs. RT compared to non-RT had lower GM volume in WB (*p* = 0.01), frontal (*p* = 0.04), left and right parietal (*p* = 0.01 and *p* = 0.03 respectively), after FDR-correction they all were at *Q* = 0.06. Decrease in eGFR and RT were associated with increase WM volume in the WB (*p* = 0.05, *Q* = 0.5 and *p* = 0.04, *Q* = 0.1)
Lijdsman, 2021^‡^	Low	Severe CKD vs. HC; 3 subgroup of CKD: dialysis, RT, pre-dialysis	T1 MRI scans with DTI-TBSS analysis were used. CKD patients had smaller nucleus accumbens volume compared to HC (*p* = 0.005; *d* = − 0.87). WM integrity in a cluster of mainly distal tracts was decreased in CKD compared to HC: increased MD (*p* = 0.018, *d* = 0.074) and decreased FA (*p* < 0.001, *d* = − 2.10). Dialysis and RT patients had smaller nucleus accumbens volume relative to HC (*p* = 0,037; *d* = − 0.94 and *p* = 0.005; *d* = − 1.28 for dialysis and RT respectively) and lower WM integrity: decreased FA and AD and increase in RD (*p* < 0.001), RT also had an increase in MD. The IFOF, ATR and SLF WM tracts contributed the most to the affected cluster. IFOF, UF, ATR and CST tracts were the most affected by CKD. Longer time since successful transplantation was significantly related to lower FA (*β* = − 0.518, *p* = 0.011)
Lijdsman, 2022^‡^	Low	Severe CKD (Stage 4 − 5)	dMRI focused on brain ROIs identified in previous work (Lijdsman, 2021). FA was positively associated with eFSIQ results in a cluster of WM tracts present across the three CKD treatment groups. Lower eFSIQ results were predicted by longer time since transplantation (SE = − 0.34; *B* = − 0.360; *P* = 0.033); lower FA and lower parental education level also predicted poorer outcomes in the identified WM cluster (*R*2 = 0.739; *p* < 0.001; *F* = 28.34). Poorer processing speed and working memory were predicted by greater exposure to dialysis and later onset of severe CKD; lower parental educational level alongside older age at diagnosis also predicted poorer outcomes (*R*^2^ = 0.455; *p* = 0.002; *F* = 8.35). Regional disruption of WM integrity (lower FA) was a mediating factor between “longer time since transplantation” and “lower intelligence”. The RRT group (dialysis and RT) had poorer eFSIQ results than the non-RRT group (pre-dialysis) (*p* = 0.030, *d* = 0.99)
Matsuda-Abedini, 2018	High	Stage 2 − 5 with CKD-non-RT, RT (transplant only) HC (controls)	Conventional brain MRI and dMRI for TBSS and ROI analysis. Macrostructural WM injury was identified in 21% (*n* = 6) with CKD vs. none in HC. TBSS analysis in CKD-non-RT vs. HC: decreased FA in both ALIC (*t* > 3, *p* = 0.05), decrease AD in left ALIC, decreased RD in left optic radiation. No differences in RT vs. HC. ROIs analysis on CKD and on RT vs. HC (independently): decrease FA with increase MD and RD in the left ALIC. ROIs analysis CKD-non-RT vs. HC: MD decrease in both PLIC, RD decrease in left PLIC, AD decrease in right PLIC. ROI analysis RT vs. HC: decrease FA in left PLIC, increase MD and RD in left optic radiation. All those findings were at a minimum of *p* < 0.05 significance
Solomon, 2021	Low	Male only CAKUT Stage 1 − 3 vs. HC	17 pre-defined ROIs were examined using MRI. Mean cerebellar GM volume was lower in the CKD group relative to HC, (*t* (38) = − 2.71, *p* = 0.01), and persisted when adjusted for prematurity in pCKD (*p* = 0.021). Mean cerebral GM volume was larger in the CKD group compared to HC, (*t* (38) = 2.08, *p* = 0.04). eGFR was a significant predictor of cerebellar GM volume in pCDK, *t*(14) = 2.21, *p* = 0.04. Within the CKD group lower cerebellar GM volume was associated with poorer verbal fluency scores (*t*(12) = 2.5, *p* = 0.03) and increase in cerebral GM volume was associated with poorer mathematics performance (*t*(12) = − 2.9, *p* = 0.01)
Steinberg, 1985	Very high	22 CRF children (residual function is < 25% of normal = Stage 4 − 5) on CAPD, HD, RT	CT scans based on children with CRF. Abnormal CT brain scans were found in 63% (*n* = 14) of patients. Cerebral atrophy was the commonest finding 59% (*n* = 13). It was mild in 9 with 1 progressing to moderate; moderate in 3 and severe in 1 patient. 75% of patients on HD had cerebral atrophy including 1 graded as severe. 50% of patients on conservative treatment and on CAPD had brain atrophy (each). Cortical infarcts were found in 2 patients, both on HD. Mean age, duration of renal failure and presence of hypertension were not significant predictor of brain findings
Valanne, 2004	High	CRF patients with RT before 5 years of age following PD, 88% with CNF	MRI was done on pCKD patient who underwent PD preceded by RT before 5 yr of age. IBZ infarcts in the periventricular WM and centrum semiovale were found in 54% (*n* = 18). They were mild in 10 Px, moderate (grade 2) in 6 Px and severe (grade 3) in 2 Px; grade 2 and 3 had cortical extensions to watershed regions. Brain lesions were positively associated with haemodynamic crises, age at first RT and dialysis time (*p* = 0.03; *p* = 0.05; *p* = 0.02 respectively). Other rare MRI findings: infarcts in the main vascular territories; cortical watershed infarcts; bilateral cerebellar WMH always in the presence of IBZ infarcts. Central and cortical atrophy in 2 and 3 patients respectively. Lesions in basal ganglia of 2 Px and caudate nucleus in 1 Px. For 26 patients (out of 33), pre-RT CT scans were available. Large infarcts and grade 3 watershed lesions were already present on CT-scans. 3 patients showed early cortical atrophy on CT scans that was not present in post-RT MRI. 1 patient had unilateral lesion pre-CT and bilateral post-MRI
van der Plas, 2022a^‡^	Low	Male only with CAKUT; Early stage: Stage 1 − 3 vs. HC	dMRI to determine WM FA. Global WM FA was reduced in CKD compared to HC (SE = − 0.38, 95% CI − 0.69 to − 0.07, FDR = 0.071, Cohen *f*^2^ = 0.46). This was underpinned by significant regional WM FA decrease in CKD compared to HC for: the body of the corpus callosum (SE = − 0.44, 95% CI − 0.75 to − 0.13, FDR = 0.04), cerebral peduncle (SE = − 0.37, 95% CI − 0.67 to − 0.07, FDR = 0.07), cingulum (hippocampus) (SE = − 0.45, 95% CI − 0.75 to − 0.14, FDR = 0.04), PLIC (SE = − 0.46, 95% CI − 0.76 to − 0.15, FDR = 0.04) as well as in the external capsule and retrolenticular internal capsule. Global WM FA was correlated to BRIEF BRI (uncorrected *p* < 0.05)
van der Plas, 2022b	Low	Male CAKUT with Stage 1 − 3 CKD vs. HC	Brain ROIs examined via MRI based on previous findings (Solomon, 2021). CKD group had an accelerated age-related increase in NfL levels compared to HC. Within the CKD group higher NfL levels were predicted by lower cerebellar gray matter (estimate = − 0.0002401; SD = 0.00007; *p* = 0.004) and reduced kidney function defined by eGFR (estimate = − 0.10; SD = 0.027; *t* (13) = − 3.65; *p* = 0.003)
Zaki, 2021	High	ESRD on HD	Evaluation of SNL using various MRI methods. SBI were present in 79.2% (*n* = 38) of the ESRD patients on HD; 45.8% (*n* = 22) had periventricular WM lesions. SBI were predicted by calcium level and dialysis duration (OR[95%CI] = 47.803 ([1.337–1708.8] *p* = 0.034; OR[95%CI] = 9.154 [1.049–79.911] *p* = 0.045 respectively). ROC curve showed that parathyroid hormone (PTH) level > 585 pg/ml, duration of dialysis > 2 years, and calcium level > 7.5 mg/dl predicted the presence of SNL (*p* = 0.025; *p* = 0.002; *p* = 0.008 respectively). All brain lesions were limited to the cerebral hemispheres with 68.4% (*n* = 26) of the patients with SBI having bilateral ones. SBI lesions ranging over 10 mm were present in 68.4% (*n* = 26) of ESRD with lesions. SWI results showed microbleeds in only 2 patients
**Functional**			
Harrell, 2021^‡^	Low	Stage 1 − 5 and subgroup analysis: moderate CKD, severe CKD	fMRI-BOLD performed during a visual-spatial working memory task. CKD and HC invoked similar brain regions. CKD compared to HC had under-activation of the posterior cingulate, anterior cingulate, precuneus and middle occipital gyrus for the encoding phase (*p* = 0.05) and in the posterior cingulate, medial frontal gyrus, occipital lobe and middle temporal gyrus for the retrieval phase (*p* < 0.05). CKD had greater activation than HC in the superior temporal gyrus, middle frontal gyrus, middle temporal gyrus, and the insula for the encoding phase (*p* = 0.05) and in the post-central gyrus for the retrieval phase (*p* < 0.05). CKD had a longer reactivity time (1725 ms vs. 2424 ms, *p* = 0.003) and a longer response time (*p* = 0.001) than HC. Overall accuracy was similar. The mean percent signal change during encoding was reduced in parietal and occipital regions. Disease severity was associated to lower mean percent signal change in parietal regions. Moderate CKD had lower mean percent signal change than severe CKD in frontal regions during retrieval phase
Herrington, 2021^‡^	High	No info on stages but mean eGFR of CKD grp is 47.6 (SD = 24.1) and subgroup analysis: disease severity score	DMN connectivity was explored via rsfMRI using the PCC for the seed-based analysis. Decrease WM connectivity in favour of HC was found in: a cluster around basal forebrain and anterior cingulate cortex (peak MNI coordinate, 4,32,0) (FWE-corrected *p* < 0.05); the right frontal pole and right superior frontal gyrus (uncorrected finding). Disease severity was associated to 2 clusters (FWE-corrected *p* < 0.05): (1) Superior frontal gyrus and frontal pole with peaks in the left paracingulate gyrus (peak MNI coordinate, − 16,14,38) and left middle frontal gyrus (− 20,40,40); (2) Left precentral and postcentral gyrus (− 62, − 8,30)
Liu, 2018b^‡^	Low	NiCK study cohort (Stage 2 − 5)	fMRI-ASL with hematocrit level-corrected blood T1. Higher uncorrected global CBF in CKD (mean = 60.2 ml/100 g/min SD = 9.0 vs. mean = 56.5 ml/100 g/min; SD = 8.0 respectively). Hct, age and sex corrected CBF in favour of CKD in: bilateral prefrontal cortices, middle and inferior temporal cortices, the posterior cingulate cortex, the precuneus, the left hippocampus, the striatum and the thalamus (*p* < 0.05). Clinical variables correlate: Age and Hct levels with CBF in WB, WM and GM for both CKD and HC groups; eGFR with CBF in GM (rho = 0.342, *p* =0.01) and WB (rho = 0.291, *p* =0.03); eGFR with Hct level (rho = 20.456, *p* < 0.0005) for the HC group only; length of disease with CBF in WB in CKD; phosphate level with CBF in GM, WM and WB; blood pressure with CBF in WM (rho = 0.244, *p* = 0.039). Cognitive function correlate: CKD patient with positive extrema had worse verbal memory outcome than non-positive extrema CKD (*p* = 0.003). CBF in precuneus and CBF of positive extrema were associated to EF deficits (rho = 0.608, *p* =0.001 and rho = 0.670 and *p* = 0.001 respectively)
Liu, 2018a	High	Any stage of CKD II–V	3 models were used to estimate blood T1 in CBF measurement via fMRI-ASL. Fixed T1 method: GM (CKD = 77.07 +/− 13.34 and HC = 68.91 +/− 10.93); WB (CKD = 65.22 +/− 10.86 and HC = 58.75 +/− 8.90) both at *p* = 0.001. Hct-based blood T1 estimation: GM (CKD = 72.47 +/− 10.63 and HC = 67.48 +/− 9.14 at *p* = 0.012); WB (CKD = 61.44 +/− 8.61 and HC = 57.55 +/− 7.27 at *p* = 0.014). Effect of Method and Method + Group on all Brain tissues *p* < 0.05. Effect of Method + Sex was significant in CBF of WB and GM. *p* < 0.05. In HC group, Hct level strongly correlated to age (rho = 0.50, *p* < 0.001)) and sex (rho = 0.38, *p* = 0.01)

Rho Spearman’s correlation test, *FEW* family wise error adjusted p values, *FDR* false discovery rate adjusted Function, *BRI *behaviour regulation index, *eFSIQ* estimated Full-scale intelligence quotient, *CAKUT* congenital anomalies *RT* renal transplant, *HC* health controls, *ESRD* end-stage renal disease, *HD* haemodialysis, *PD* peritoneal dialysis, Px patient, *ROI* regions of interest, *ASL* arterial spin labelling, *BOLD* blood oxygen level tomography, *dMRI* diffusion *MRI*, *fMRI* functional *MRI*, *WM* white matter, *WB* whole brain, *GM* gray matter, axial diffusivity, *RRT* renal replacement therapy, *ALIC* anterior limb of internal capsule, *PLIC* posterior limb infarcts, *WMH* white matter hyperintensities, *cGMP* cyclic guanosine monophosphate, *DMN* default mode network, *Hct* haematocrit, *eGFR* estimated glomerular filtration rate, *EF* executive functions, *CAPD* continuous ambulatory peritoneal dialysis

## Results

**Fig. 1:**
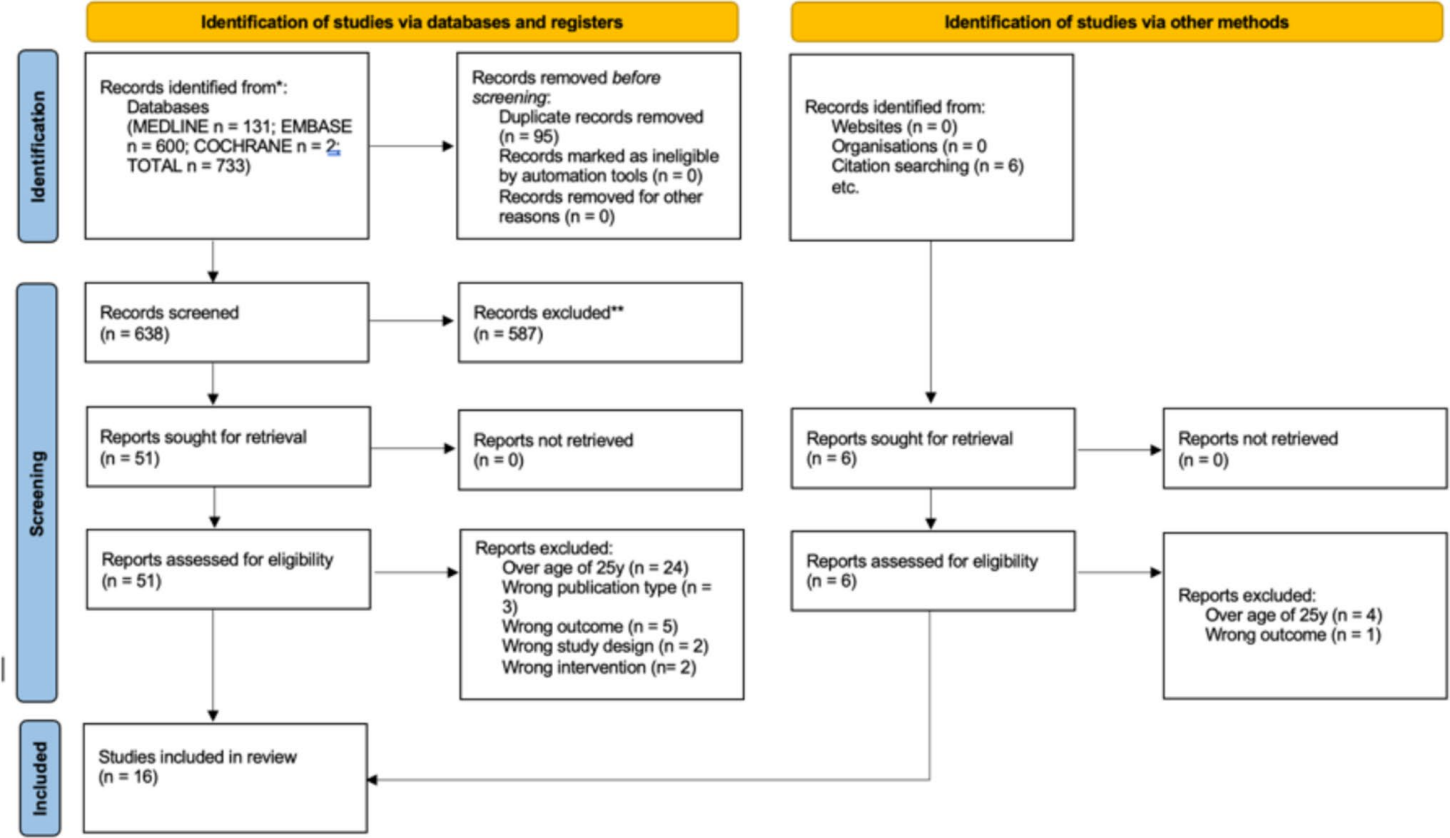
Preferred reporting items for systematic review and meta-analysis (PRISMA) flowchart [24]

Following deduplication, 638 articles were identified with six more on citation searching. After the staged screening, 16 articles met eligibility criteria (Fig. 1) totalling 737 individuals (CKD, *n* = 463; Healthy controls (HC), *n* = 274). Fifteen studies were cross-sectional (ten with a control group), and one longitudinal (no control group).

### Quality assessment results

NOS [26] was used for quality assessment of the longitudinal study and a modified NOS [27] for the cross-sectional studies. A summary of the results is reported in *Table* S3. Most studies were small. Seven studies were assessed as low risk of bias (Table S3).

### Study characteristics

Study characteristics are reported in Table 1. Studies were published between 1985 and 2022, and mostly from the USA (*n* = 9). Four studies used functional imaging: functional magnetic resonance (fMRI) – arterial spin labelling (fMRI-ASL) (*n* = 2) and fMRI – blood oxygen level dependent (fMRI-BOLD) (*n* = 2). Twelve studies used structural imaging, including computerized tomography (CT) (*n* = 4), diffusion MRI (dMRI) (*n* = 4) and conventional MRI (*n* = 14). Three papers focused exclusively on males with congenital anomalies of the kidney and urinary tract (CAKUT).

### Summary of study results

#### Structural neuroimaging

**Brain volume** was evaluated in six studies with total CKD patients (*n* = 161) and HC (*n* = 45) [29–34]. Historical CT studies from the 1980 s and 1990 s [33, 34] reported brain atrophy in 23–59% of paediatric CKD patients, though these findings must be interpreted cautiously given methodological limitations of the era.

Lijdsman [29] reported smaller subcortical grey matter (GM) volume in the nucleus accumbens limited to post-kidney replacement therapy (KRT) patients with CKD stages 4–5 when compared to HC. This was more pronounced in kidney transplant (KT) patients, though prior dialysis in some KT patients may have influenced the finding. No association with estimated glomerular filtration rate (eGFR) was found.

In one CKD population stages 2–5 (48% CKD 3–CKD5), Hartung [30] found no differences in whole brain or regional GM volume in models accounting for age and sex.

Solomon [31] performed a cross-sectional study with males (mean age of 12 years) having early CKD (eGFR > 30 ml/min/1.73 m^2^) due to CAKUT with no history of dialysis and reported a paradoxical increase in cerebral GM volume associated with poorer mathematics outcomes. Lower cerebellar GM volume was predicted by decreases in eGFR and was associated with poorer performances in verbal fluency.

Van der Plas [32] followed the same cohort [31] to identify the association between markers of neuroinjury and brain volumes. Here, cerebellar GM volume and eGFR (in the CKD group) were inversely associated with neuroaxonal injury (plasma neurofilament light chain (NfL) concentrations). Patients with CKD demonstrated a faster age-related increase in NfL concentrations than HC.

**White matter abnormalities** in CKD were reported in four studies [29, 35–37] with total CKD patients (*n* = 74) and HC (*n* = 61). Four studies consistently demonstrated white matter impairment across CKD stages using diffusion MRI techniques.

Lijdsman et al. [29] found widespread decreases in fractional anisotropy (FA) (a measure of white matter integrity) in patients with CKD stages 4–5, with the most severe changes occurring post-transplant. The duration since transplantation was inversely associated with white matter integrity. Those brain regions were further analysed alongside neurocognitive outcomes [36]. Only one cluster showed positive association between FA and a lower estimated intelligence quotient (IQ). Factors such as time since KT, dialysis duration and a later CKD diagnosis were negatively associated with cognitive outcomes, including IQ, processing speed and working memory. Parental education appeared protective, while a later onset of severe CKD predicted poorer cognitive outcomes.

Even in early-stage CKD, van der Plas et al. [35] demonstrated global and regional white matter impairment in pre-transplant patients with CAKUT. Major white matter tracts including the corpus callosum and posterior limb of the internal capsule showed decreased integrity, though no association with eGFR was found. However, the interpretation of disease progression effects is limited as the duration of specific eGFR levels in participants was not reported.

Matsuda-Abedini et al. [37] studied a heterogeneous group of 29 children with CKD (stages 2–5) and found white matter injuries exclusively in CKD patients compared to controls. The anterior limb of the internal capsule was particularly affected, showing consistent changes across multiple diffusion parameters.

**White matter hyperintensities** were reported by three studies (all high-risk bias), only looking at patients with CKD (*n* = 91) [38–40]. All the studies were assessed as “High-Risk” bias partly due to the potential for interactions with other contributing factors including vascular risks and prematurity.

Zaki et al. [38] studied HD patients (mean age 20 years). Seventy-nine percent had SBI limited to the cerebrum suggestive of axonal damage and demyelination. They commonly occurred bilaterally (68%) and mainly within frontal/temporal lobes. Hyperparathyroidism and higher serum calcium concentrations (even within the normal range) were associated with SBI occurrence.

Valanne et al. [39] examined patients post-KT preceded by peritoneal dialysis (PD) (88% Finnish-type congenital nephrotic syndrome (CNF), mean age 8 years). Fifty-four percent had deep WM lesions in internal border zones, indicating impairment of small vessels including deep perforating arteries and penetrating branches from major cerebral arteries. Haemodynamic instability was a risk factor for ischemic lesions. GM and WM volume was reduced. Early KT appeared to improve neurological function and protect against lesions.

Carlström et al. [40] reviewed mixed CT/MRI imaging of patients undergoing PD (median age 0.22 years). Findings were reviewed in correlation with nitrate and nitrite concentrations, and blood pressure. Nitric oxide bioavailability dysregulation and persistent low systolic blood pressure were potentially related to ischemic lesions. Brain findings were numerous but were limited to cerebrum and mostly within the occipital lobe.

#### Functional neuroimaging

**Functional connectivity** was assessed by two studies with total CKD patients (*n* = 88) and HC (*n* = 69) [41, 42].

While both studies used fMRI-BOLD, Harrell et al. [41] assessed neural activation during a delayed-response visual memory task in CKD stages 2–5 (mean age 14.4 years). Similar brain regions (prefrontal cortex, premotor cortex, parietal regions and occipital) were activated in both CKD and HC with no differences in overall accuracy being reported. However, CKD had longer reactivity and response times to visual stimuli. In CKD, key regions of the default-mode-network (DMN) showed reduced activation during both the encoding and retrieval phases (posterior cingulate cortex (PCC), precuneus and medial frontal gyrus). Lower neural engagement was noted in regions essential for attention: anterior cingulate cortex (ACC), visual/spatial encoding and retrieval (occipital lobe and PCC) and mental imagery (precuneus).

Herrington et al. [42] (mean eGFR = 48 ml/min/1.73 m^2^; mean age 16 years); used resting-state fMRI to examine DMN connectivity, finding decreased connectivity in the anterior cingulate cortex regardless of treatment status. Functional connectivity correlated with disease severity in frontal regions, with no areas showing increased connectivity in patients with CKD.

Two studies examined **cerebral blood flow** (CBF) using arterial spin labelling, with total CKD patients (*n* = 73) and HC (*n* = 57) [43, 44]. Liu et al., in patients with CKD stages 2–5 (mean age 14.5 years), found increased grey matter and whole brain blood flow, though results varied depending on the method used to correct for blood T1 relaxation time. When corrected for hematocrit, sex and age, the DMN showed increased blood flow, particularly in the PCC and precuneus.

Importantly, increased CBF in CKD patients was associated with EF deficits and, positive CBF extrema to poorer verbal memory performance. This suggests a pathological rather than compensatory response.

## Discussion

This is the first systematic review of neuroimaging studies looking exclusively at brain studies in CKD using clear age cutoffs. Despite limited and heterogenous data, evidence suggests that CKD may play a role both in brain injury and disruption of neurodevelopment. However, we must also recognize the interplay between developmental drivers of disease in tandem with clinical factors of CKD.

Our systematic review identified several potential mechanisms underlying brain changes in CKD. Vascular mechanisms, including haemodynamic instability, hypertension and altered cerebrovascular autoregulation, appear to contribute to white matter injury and infarcts. Metabolic factors such as anaemia, bone disease markers (calcium, parathyroid hormone) and uremic toxins show potential associations with brain abnormalities across multiple studies. Treatment effects also play a significant role, with both dialysis and transplantation demonstrating specific patterns of brain injury, although early transplantation may be protective for some outcomes. Finally, developmental factors related to the timing of CKD onset relative to critical neurodevelopmental periods may influence both the pattern and severity of brain changes, highlighting the unique vulnerability of the paediatric population.

### Structural studies

The structural imaging findings reveal a concerning pattern of brain abnormalities beginning in early-stage CKD and progressing with disease severity. White matter changes appear to be the most consistent finding, present even in mild disease and detectable using sensitive diffusion MRI techniques [29, 35–37]. The specific white matter tracts affected—including the corpus callosum, internal capsule and thalamocortical projections—are critical for cognitive functions known to be impaired in CKD, particularly attention, working memory and processing speed [45, 46].

The high prevalence of white matter hyperintensities (54–79%) in patients on dialysis [38, 39] is particularly alarming, as these lesions are associated with increased stroke risk and cognitive decline in adult populations [47]. The identification of modifiable risk factors including serum calcium levels, parathyroid hormone concentrations and haemodynamic instability suggests potential targets for intervention.

Brain volume changes showed more variable results, possibly reflecting differences in study populations, disease severity and methodological approaches. The paradoxical increase in grey matter volume reported in one CAKUT study [31] may reflect altered developmental trajectories, as normal brain maturation involves synaptic pruning and volume reduction in certain regions [48].

### Functional studies and cognitive correlates

The functional changes [41, 42] described in this systematic review align well with established patterns of cognitive impairment in paediatric CKD, supporting the hypothesis that observed neuroimaging abnormalities have direct clinical and functional relevance [49]. Functional imaging studies suggest a potential association between identified abnormalities in the DMN and regions involved in executive function. The DMN, active during rest and introspective tasks, shows altered connectivity in numerous neuropsychiatric conditions [50] and may represent a common pathway for CKD-related cognitive impairment [2, 51–53]. Although studies [41, 42] lacked cognitive correlates, affected regions map to attention, working memory and EF, in line with previous paediatric CKD [3, 5–7, 54] and adult CKD [55–58] literature.

CKD significantly affects CBF, with studies demonstrating an inverse relationship between haematocrit and CBF [43, 44]. Paradoxically, increased CBF was associated with worse cognitive performance, suggesting impaired cerebrovascular autoregulation rather than beneficial physiological compensation. Anaemia, which is common in CKD [59] and known to affect cognition [60], may initially trigger compensatory increases in CBF to maintain adequate cerebral oxygen delivery [61]. However, this compensatory mechanism appears to become maladaptive, potentially impairing autoregulation and compromising blood–brain barrier integrity. These CBF abnormalities were specifically associated with verbal memory and executive function deficits [44]. Notably, in the prefrontal cortex and key DMN regions, CBF increases occurred independently of hematocrit levels, suggesting that these brain areas may be particularly vulnerable to CKD-related vascular dysfunction.

## Limitations

Several limitations must be acknowledged. Most studies were cross-sectional with small sample sizes, preventing assessment of causality or developmental trajectories and limiting statistical power. The heterogeneity of CKD aetiologies, disease stages and treatments across studies made the identification of consistent patterns or mechanisms more challenging. Altogether, this restricts the generalisability of the results. Those limitations led us to conduct a synthesis without meta-analysis, but as higher-quality, more homogeneous paediatric studies emerge, a meta-analysis will be highly valuable. Lastly, to enhance relevance to young CKD patients, we applied strict age criteria, which reduced the number of eligible studies and may have excluded relevant data.

### Concluding remarks

Understanding the mechanisms underlying brain abnormalities in paediatric CKD is crucial for developing targeted therapies and optimizing long-term neurodevelopmental outcomes in this vulnerable population. This systematic review provides compelling evidence that CKD affects brain structure and function across all disease stages in children and young adults. Multiple cognitive manifestations in CKD were associated with structural or functional brain changes, or both. SBI which are associated with stroke risk and cognitive decline are commonest in the elderly population but were reported in concerningly high proportion in young CKD participants. Insufficient high-quality studies are available, but in more recent reports an effort to study more homogeneous cohorts has been made.

This review underscores the need for larger, longitudinal studies in well-defined paediatric CKD populations to more fully ascertain the impact of CKD on neurodevelopment. 

## Supplementary Information

Below is the link to the electronic supplementary material.

ESM 1 Graphical abstract (PPTX 655 KB)

ESM 2 (DOCX 547 KB)

ESM 3 (DOCX 271 KB) 

ESM 4 (XLSX 34.7 KB)

## Data Availability

All data underlying the findings of this study are fully reported within the article, its supplementary materials and the cited sources.
