# Supplementary material for: Systematic review of neuroimaging findings in children and young adults with chronic kidney disease
Source: Pediatr Nephrol. 2025 Dec 11;41(8):2425–41. doi: 10.1007/s00467-025-07094-5 (PMC13337593; doi:10.1007/s00467-025-07094-5)
Supplement: Supplementary file 1 — Graphical abstract (PPTX 655 KB) [file 467_2025_7094_MOESM1_ESM.pptx]

## Slide 1
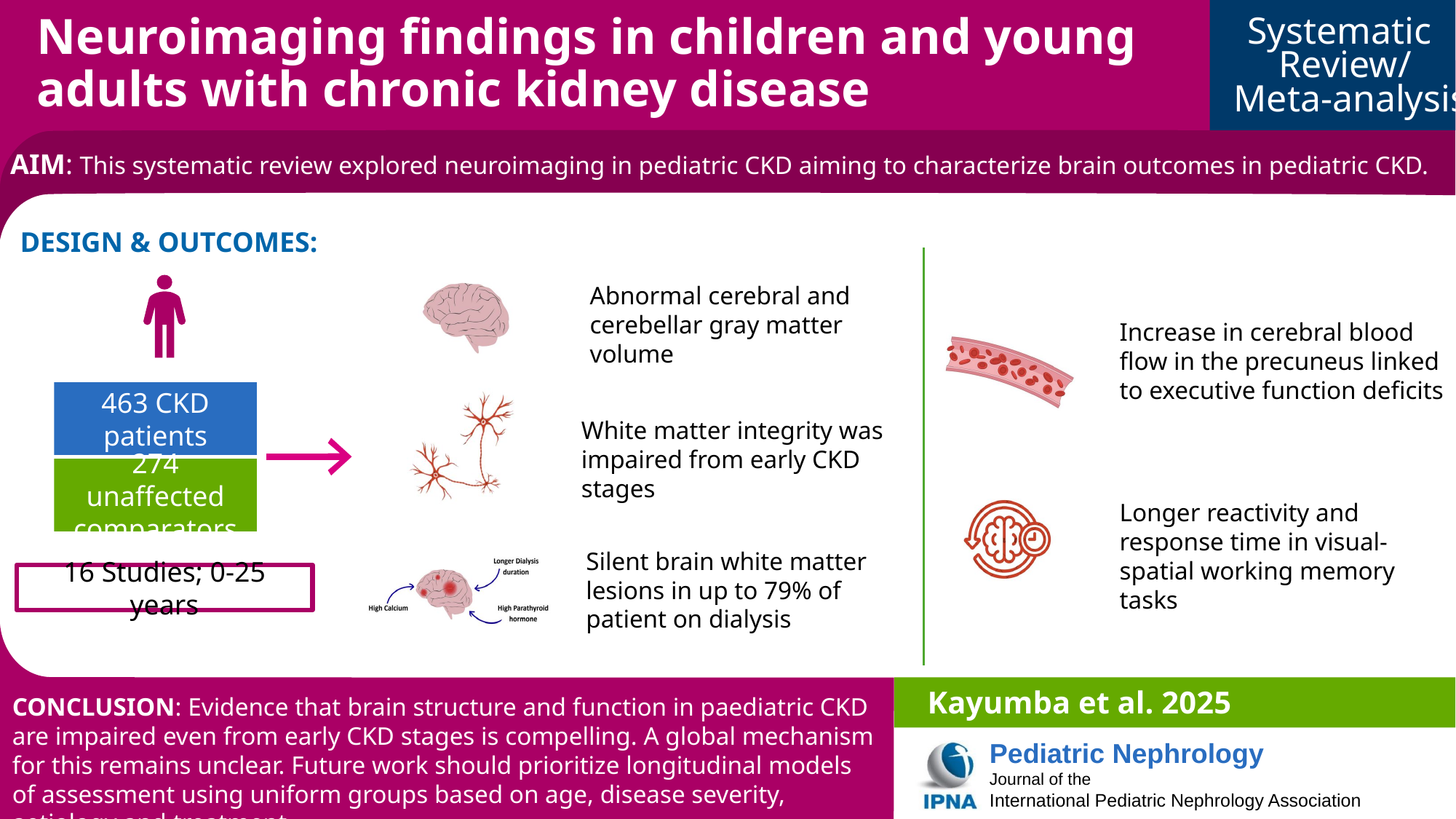

Neuroimaging findings in children and young adults with chronic kidney disease
AIM: This systematic review explored neuroimaging in pediatric CKD aiming to characterize brain outcomes in pediatric CKD.
DESIGN & OUTCOMES:
Abnormal cerebral and cerebellar gray matter volume
Increase in cerebral blood flow in the precuneus linked to executive function deficits
White matter integrity was impaired from early CKD stages
Longer reactivity and response time in visual-spatial working memory tasks
Silent brain white matter lesions in up to 79% of patient on dialysis
463 CKD patients
274 unaffected comparators
16 Studies; 0-25 years
Kayumba et al. 2025
CONCLUSION: Evidence that brain structure and function in paediatric CKD are impaired even from early CKD stages is compelling. A global mechanism for this remains unclear. Future work should prioritize longitudinal models of assessment using uniform groups based on age, disease severity, aetiology and treatment.
