# Supplementary material for: Systematic review of neuroimaging findings in children and young adults with chronic kidney disease
Source: Pediatr Nephrol. 2025 Dec 11;41(8):2425–41. doi: 10.1007/s00467-025-07094-5 (PMC13337593; doi:10.1007/s00467-025-07094-5)
Supplement: Supplementary file 2 — (DOCX 547 KB) [file 467_2025_7094_MOESM2_ESM.docx]

## Appendix 1. PRISMA-P Protocol (using the same information as in the PROSPERO application)

## Appendix 2. Entries from the Data extraction table

General study information including

- Title
- Study ID
- Publication type
- Study design
- Where and Language

Study background

- Aims and objectives
- Sample
- Duration

Population

- Inclusion criteria
- Exclusion criteria
- Age means (range)
- Gender (nM/nF)
- Ethnicity
- Co-morbidities
- Socio-demographic/Clinical parameters
- Treatment method
- CKD stage
- Duration means (range)
- Aetiology

Comparator

- Description

Intervention

- Imaging method used
- Procedure
- Method of analyses
- Notes

Main outcome

- Biochemical data
- Outcome
- Results
- Statistical analysis
- Notes 2

Additional outcomes

- Secondary outcome
- Secondary results
- Statistical analysis 2
- Notes 3
- Additional outcomes
- Additional results
- Notes 4

Extra

- Tables and Graph 1
- Tables and Graph 2

Brief quality assessment

- Statistical analysis reliability
- Study funding
- Limitations
- Baseline imbalances (yes or no)

**Appendix 3. Newcastle-Ottawa Scale for cross-sectional studies and for cohort studies**

^^

^^
